# The genetic network underlying the evolution of pathogenicity in avian *Escherichia coli*

**DOI:** 10.3389/fvets.2023.1195585

**Published:** 2023-06-21

**Authors:** Nicola Palmieri, Ilias Apostolakos, Surya Paudel, Michael Hess

**Affiliations:** ^1^Clinic for Poultry and Fish Medicine, Department for Farm Animals and Veterinary Public Health, University of Veterinary Medicine, Vienna, Austria; ^2^Veterinary Research Institute, ELGO-DIMITRA, Thessaloniki, Greece; ^3^Department of Infectious Diseases and Public Health, Jockey Club College of Veterinary Medicine and Life Sciences, City University of Hong Kong, Kowloon, Hong Kong SAR, China

**Keywords:** GWAS, APEC, pathogenicity (infectivity), *Escherichia coli* (*E. coli*), protein–protein interaction (PPI) network

## Abstract

**Introduction:**

Colibacillosis is a worldwide prevalent disease in poultry production linked to *Escherichia coli* strains that belong to the avian pathogenic *E. coli* (APEC) pathotype. While many virulence factors have been linked to APEC isolates, no single gene or set of genes has been found to be exclusively associated with the pathotype. Moreover, a comprehensive description of the biological processes linked to APEC pathogenicity is currently lacking.

**Methods:**

In this study, we compiled a dataset of 2015 high-quality avian *E. coli* genomes from pathogenic and commensal isolates, based on publications from 2000 to 2021. We then conducted a genome-wide association study (GWAS) and integrated candidate gene identification with available protein-protein interaction data to decipher the genetic network underlying the biological processes connected to APEC pathogenicity.

**Results:**

Our GWAS identified variations in gene content for 13 genes and SNPs in 3 different genes associated with APEC isolates, suggesting both gene-level and SNP-level variations contribute to APEC pathogenicity. Integrating protein-protein interaction data, we found that 15 of these genes clustered in the same genetic network, suggesting the pathogenicity of APEC might be due to the interplay of different regulated pathways. We also found novel candidate genes including an uncharacterized multi-pass membrane protein (yciC) and the outer membrane porin (ompD) as linked to APEC isolates.

**Discussion:**

Our findings suggest that convergent pathways related to nutrient uptake from host cells and defense from host immune system play a major role in APEC pathogenicity. In addition, the dataset curated in this study represents a comprehensive historical genomic collection of avian *E. coli* isolates and constitutes a valuable resource for their comparative genomics investigations.

## Introduction

*Escherichia coli* infection in poultry might cause a wide variety of pathologies such as salpingitis, peritonitis, airsacculitis, femoral head necrosis, cellulitis or omphalitis, which are collectively called colibacillosis (1). This disease is one of the major concerns in poultry production due to high economic losses and the requirement of antibiotic use for their treatment, which has public health importance as well. In recently published prevalence studies in broiler and layer chickens, *E. coli* was found to be the leading pathogen (2, 3). Despite being one of the major diseases in poultry, options for prevention by vaccination are still limited largely due to the high genomic heterogeneity and complex pathogenicity mechanisms of avian *E. coli* isolates (4). Although colibacillosis might exhibit varying clinical manifestations in different avian species (1), the underlying molecular mechanisms of pathogenicity are thought to be common (2, 3).

Historically, isolates collected from the systemic organs of diseased birds are classified as avian pathogenic *E. coli* (APEC), a subset of extra-intestinal pathogenic *E. coli* (ExPEC) responsible for causing colibacillosis. In contrast, commensal strains originating from the gut are classified as avian fecal *E. coli* (AFEC). APEC strains can arise through four distinct evolutionary mechanisms (5): (1) the extra-intestinal spread of pathogenic clones possessing specific virulence factors; (2) host-mediated selection that favors highly pathogenic strains; (3) plasmid-mediated extra-intestinal spread, which involves the transfer of plasmids among lineages; and (4) horizontal gene transfer that generates novel APEC strains through the reassortment of plasmids and virulence factors.

However, the genetic distinction between APEC and AFEC remains unclear, as researchers have yet to define a unique set of features that can reliably identify APEC strains. Numerous attempts have been made to pinpoint marker genes specific to APEC strains [e.g., (6–8)]. The most widely used typing method focuses on five genes (*iutA, hlyF, iss, iroN, ompT*) (7), which are located on the ColV plasmid (9). This method has been employed to characterize APEC strains in over 60 genomic reports. However, a large-scale study involving 568 isolates questioned the discriminatory power of these five genes, revealing their widespread presence in commensal isolates as well (5). Additionally, a recent analysis of 3,479 isolates demonstrated that no single gene can be uniquely associated with APEC strains (8). Consequently, high-resolution studies are necessary to uncover the intricate genomic traits that contribute to avian *E. coli* pathogenicity.

A promising approach to unravel the genetic basis of the APEC pathotype is the Genome-Wide Association Study (GWAS). A recent GWAS analysis, involving 568 *E. coli* isolates from healthy and diseased birds on commercial poultry farms across various European regions, identified 143 APEC-associated genes. These genes play roles in metabolism, lipopolysaccharide synthesis, heat shock response, antimicrobial resistance, and toxicity (5). However, a simulation study demonstrated that the power of detecting associations in bacterial GWAS significantly increases as the sample size approaches 1,000 genomes, reaching a plateau at around 3,000 genomes (10). In light of this information, we conducted an extensive literature and database search to collect all available avian *E. coli* genomes. This effort resulted in a comprehensive dataset comprising 2,015 high-quality genomes. We then integrated GWAS analysis with existing protein–protein interaction data to identify candidate genes associated with the APEC pathotype. This approach allowed us to propose a systematic overview of the cellular processes underlying APEC pathogenicity.

## Methods

### Generation of the avian *E. coli* genomic database

Literature screening was performed to collect studies that reported genome sequences of *E. coli* isolated from domestic avian species (chickens, turkeys, ducks, and geese). The search was focused on field studies where (a) commensal *E. coli* retrieved from the intestinal tract of apparently healthy birds (AFEC) and (b) pathogenic isolates retrieved from clinical cases of avian colibacillosis (APEC) were reported. Other types of studies such as those describing experimental infection models were not included. Literature searches were performed in the PubMed and Web of Science databases during November-December 2021, using the following Boolean search string:

(chicken^*^ OR poultry^*^ OR broiler^*^ OR turkey^*^ OR hen^*^ OR fowl^*^ OR geese OR goose OR duck^*^) AND (“*Escherichia coli*” OR “*E. coli*” OR APEC OR “pathogenic E. coli” OR “pathogenic Escherichia coli” OR ExPEC OR colibacillosis OR colisepticaemia OR colisepticemia OR peritonitis OR salpingitis OR cellulitis) AND (genom^*^ OR wgs)

Studies published before 2000 or written in languages other than in English were excluded. The studies (*n* = 730) meeting the above set criteria were downloaded, uploaded to the Rayyan systematic review program (11) and deduplicated. In the first phase, studies were screened at the title and abstract level, and those not complying with our inclusion criteria were excluded. In the second phase, studies passing phase one and those marked as unclear were evaluated at the full-text level. Ultimately, 48 studies reporting 2,209 *E. coli* genomes fulfilled the eligibility criteria and were included in our analyses.

From each publication, the corresponding genome assemblies were downloaded from the NCBI assembly database based on the accession numbers provided. If genome sequences were not available, reads data from the NCBI SRA database were assembled using the Comprehensive Genome Analysis service from the PATRIC web application (12) with default parameters (minimum contig length = 300 bp) by directly providing the SRA accession to the PATRIC interface. The quality of the assemblies was assessed with the checkM software using the default settings (13). Low-quality genomes (*n* = 185) were filtered out based on the following criteria: completeness ≤ 95%, contamination ≥ 5%, number of contigs ≥ 800, N50 < 20 Kbp and genomes with no marker genes detected. Moreover, genomes were annotated with PROKKA (14) using the default parameters. Finally, *E. coli* phylogroups and multi-locus sequence types (MLSTs) were computed with the ClermontTyping tool (15) and the pubMLST (https://pubmlst.org/) database, respectively. In addition to the genome sequences, a metadata table describing the 2015 high-quality isolates was generated (Supplementary Table 1).

### Gene-based, SNP-based and unitigs-based GWAS

A schematic representation of the pipeline is summarized in Supplementary Figure 1. Initially, a gene presence/absence matrix for the 2015 high-quality genomes was produced using Panaroo (16) (parameters –clean-mode strict -a core –aligner mafft –core_threshold 0.95) by providing the gene annotations from PROKKA as input (14). Next, a phylogenetic tree was generated using FastTree (17) (parameters -nt -nopr -cat 20 -nosupport -fastest) from the Panaroo core-gene alignment. For the gene-based GWAS, the gene presence/absence matrix and phylogenetic tree were used as input to the R package treeWAS (18) (parameters *p*-value = 0.05), together with a phenotype binary vector in which the phenotype of the 2015 strains was encoded with 0 for AFEC and 1 for APEC. The program returned a list of genes that were significantly enriched in one class over the other. For each of the significant genes, its prevalence (in percentage) among APEC and AFEC isolates was calculated. Then, significant genes with prevalence > 50% in APEC and APEC vs. AFEC prevalence ratio > 1 were retained for further processing. For the SNP-based GWAS, the 2015 strains were aligned with parsnp (19) (default parameters) using the APEC O1 strain as a reference (GenBank accession GCA_902880315.1). From the parsnp output, a VCF file with SNPs was generated and separated into biallelic SNPs and multiallelic SNPs using bcftools (20) (parameters view m2 -M2). Biallelic SNPs were filtered by removing sites where the alternative allele was equal to N and converted to a binary matrix, encoding the reference allele as 1 and the alternative allele as 0; finally, each SNP was named using the convention snp_xxxxxx where xxxxxx is the genomic position in the parsnp alignment. Multiallelic SNPs were also filtered by removing sites where one of the alternative alleles was equal to N and converted into a binary matrix using a multi-line representation, following the suggestions from Saund et al. (21). Recoded multiallelic SNPs were named using the convention snp_xxxxxx_y where xxxxxx is the genomic location in the parsnp alignment and y is a progressive number (1, 2, 3) uniquely identifying the alternative allele. The whole set of biallelilc SNPs and recoded multiallelic SNPs was given as input to treeWAS (parameters *p*-value = 0.05) together with the phylogenetic tree and phenotype binary vector that were used for the gene-based GWAS. The program returned a list of significant SNPs associated to APEC isolates. The corresponding gene associated to each significant SNP was retrieved from the parsnp output. To further validate the significant genes obtained from both gene-based and SNP-based GWAS, we conducted a unitig-based GWAS. We generated unitigs for all genomes with unitig caller (4) (default parameters) and assessed their association with the APEC phenotype using the same approach as in the previous GWAS methods. We then aligned the significant unitigs to the candidate genes from the gene-based and SNP-based GWAS analyses using BLASTN (parameters -word_size 7 -evalue 1e-05). Only candidate genes with at least 5 aligned unitigs were retained as final candidates, ensuring a more robust validation of the identified genes.

## Results

### Generation of the avian *E. coli* genomic database

Through a comprehensive literature screening, we identified an initial set of 730 publications reporting *E. coli* genomes from different avian hosts. After deduplication and extensive manual curation, we refined our list to 48 publications that all together describe the genome sequences of 2209 avian *E. coli* genomes divided into APEC and AFEC isolates. After filtering out low quality genomes, we generated a final database of 2015 *E. coli* avian genomes including 1,089 APEC isolates and 926 AFEC isolates. Host distribution for this dataset includes chicken (*n* = 1,732), turkey (*n* = 152), duck (*n* = 56), wildfowl (*n* = 12), gull (*n* = 5), goose (*n* = 4) and poultry unknown (*n* = 54). A summary table containing the number of isolates in each study and the corresponding publication reference is shown in Table 1, while the metadata table for the 2015 isolates is provided in Supplementary Table 1.

**Table 1:** List of publications used to construct the avian *E. coli* genomic database.

**Publication**	**Pathotype(s)**	**No. isolates**	**References**
2012_Rojas	APEC	1	Rojas TCG, Parizzi LP, Monique RT, Chen L, Pereira GAG, Sangal V, et al. Draft genome of a Brazilian avian-pathogenic *Escherichia coli* strain and *in silico* Characterization of virulence-related genes. J Bacteriol. 2012;194: 3023–3025. doi: 10.1128/JB.00394-12
2013_Dziva	APEC	2	Dziva F, Hauser H, Connor TR, van Diemen PM, Prescott G, Langridge GC, et al. Sequencing and functional annotation of avian pathogenic *Escherichia coli* serogroup O78 strains reveal the evolution of E. coli lineages pathogenic for poultry via distinct mechanisms. Infect Immun. 2013;81: 838–849. doi: 10.1128/IAI.00585-12
2013_Rojas	APEC	3	Rojas TCG, Maluta RP, Parizzi LP, Koenigkan LV, Yang J, Yu J, et al. Genome sequences of avian pathogenic *Escherichia coli* strains isolated from Brazilian commercial poultry. Genome Announc. 2013;1. doi: 10.1128/genomeA.00110-13
2014_deBeen	AFEC	4	de Been M, Lanza VF, de Toro M, Scharringa J, Dohmen W, Du Y, et al. Dissemination of Cephalosporin Resistance Genes between *Escherichia coli* Strains from Farm Animals and Humans by Specific Plasmid Lineages. PLOS Genet. 2014;10.
2014_Ge	APEC	1	Ge XZ, Jiang J, Pan Z, Hu L, Wang S, Wang H, et al. Comparative genomic analysis shows that avian pathogenic *Escherichia coli* isolate IMT5155 (O2:K1:H5; ST complex 95, ST140) shares close relationship with ST95 APEC O1:K1 and human ExPEC O18:K1 strains. PLoS One. 2014;9: e112048. doi: 10.1371/journal.pone.0112048
2015_Giufre	AFEC+APEC	2	Maria G, Maria A, Caterina G, Luca B, Marina C. Whole-Genome Sequences of Multidrug-Resistant *Escherichia coli* Strains Sharing the Same Sequence Type (ST410) and Isolated from Human and Avian Sources in Italy. Genome Announc. 2015;3. Available: https://pubmed.ncbi.nlm.nih.gov/26159534/
2015_Huja	APEC	1	Huja S, Oren Y, Trost E, Brzuszkiewicz E, Biran D, Blom J, et al. Genomic avenue to avian colisepticemia. MBio. 2015;6. doi: 10.1128/mBio.01681-14
2016_Cordoni	APEC	89	Cordoni G, Woodward MJ, Wu H, Alanazi M, Wallis T, La Ragione RM. Comparative genomics of European avian pathogenic *E. coli* (APEC). BMC Genomics. 2016;17. doi: 10.1186/s12864-016-3289-7
2016_Ewers	APEC	1	Ewers C, Göttig S, Bülte M, Fiedler S, Tietgen M, Leidner U, et al. Genome sequence of avian *Escherichia coli* strain IHIT25637, an extraintestinal pathogenic *E. coli* strain of ST131 encoding colistin resistance determinant MCR-1. Genome Announc. 2016;4. doi: 10.1128/genomeA.00863-16
2016_Maluta	APEC	1	Maluta RP, Nicholson B, Logue CM, Nolan LK, Rojas TCG, da Silveira WD. Complete genomic sequence of an avian pathogenic *Escherichia coli* strain of serotype O7:HNT. Genome Announc. 2016;4. doi: 10.1128/genomeA.01611-15
2016_Nicholson	APEC	1	Nicholson BA, Wannemuehler YM, Logue CM, Li G, Nolan LK. Complete genome sequence of the avian-pathogenic *Escherichia coli* strain APEC O18. Genome Announc. 2016;4. doi: 10.1128/genomeA.01213-16
2016_Ronco	APEC	2	Ronco T, Stegger M, Andersen PS, Pedersen K, Li L, Thøfner ICN, et al. Draft genome sequences of two avian pathogenic *Escherichia coli* strains of clinical importance, E44 and E51. Genome Announc. 2016;4. doi: 10.1128/genomeA.00768-16
2016_Wang	APEC	1	Wang X, Wei L, Wang B, Zhang R, Liu C, Bi D, et al. Complete genome sequence and characterization of avian pathogenic *Escherichia coli* field isolate ACN001. Stand Genomic Sci. 2016;11: 1–7. doi: 10.1186/s40793-015-0126-6
2017_Jorgensen	APEC	1	Jørgensen SL, Kudirkiene E, Li L, Christensen JP, Olsen JE, Nolan L, et al. Chromosomal features of *Escherichia coli* serotype O2: K2, an avian pathogenic *E. coli*. Stand Genomic Sci. 2017;12: 1–9. doi: 10.1186/s40793-017-0245-3
2017_Kolsut	APEC	12	Kołsut J, Borówka P, Marciniak B, Wójcik E, Wojtasik A, Strapagiel D, et al. *In silico* analysis of virulence associated genes in genomes of *Escherichia coli* strains causing colibacillosis in poultry. J Vet Res. 2017;61: 421–426. doi: 10.1515/jvetres-2017-0051
2017_Ronco	AFEC+APEC	106	Ronco T, Stegger M, Olsen RH, Sekse C, Nordstoga AB, Pohjanvirta T, et al. Spread of avian pathogenic *Escherichia coli* ST117 O78:H4 in Nordic broiler production. BMC Genomics. 2017;18. doi: 10.1186/s12864-016-3415-6
2017_Wang	AFEC	99	Wang Y, Zhang RM, Li JY, Wu ZW, Yin WJ, Schwarz S, et al. Comprehensive resistome analysis reveals the prevalence of NDM and MCR-1 in Chinese poultry production. Nat Microbiol. 2017;2.
2018_Alba	AFEC	28	Alba P, Leekitcharoenphon P, Franco A, Feltrin F, Ianzano A, Caprioli A, et al. Molecular Epidemiology of mcr-Encoded Colistin Resistance in Enterobacteriaceae From Food-Producing Animals in Italy Revealed Through the EU Harmonized Antimicrobial Resistance Monitoring. Front Microbiol. 2018;9.
2018_Chen	APEC	10	Li C, Leyi W, Afrah Kamal Y, Jilei Z, Jiansen G, Kezong Q, et al. Genetic characterization of extraintestinal *Escherichia coli* isolates from chicken, cow and swine. AMB Express. 2018;8: 117. Available: https://pubmed.ncbi.nlm.nih.gov/30019301/
2018_Falgenhauer	AFEC	79	Linda F, Can I, Kwabena O, Charity Wiafe A, Benedikt H, Ralf K, et al. Detection and Characterization of ESBL-Producing *Escherichia coli* From Humans and Poultry in Ghana. Front Microbiol. 2018;9: 3358. Available: https://pubmed.ncbi.nlm.nih.gov/30697208/
2018_Nielsen	APEC	1	Nielsen DW, Mangiamele P, Ricker N, Barbieri NL, Allen HK, Nolan LK, et al. Complete Genome Sequence of Avian Pathogenic *Escherichia coli* Strain APEC O2-211. Microbiol Resour Announc. 2018;7. doi: 10.1128/mra.01046-18
2018_Poulsen	APEC	8	Louise Ladefoged P, Magne B, Steffen Lynge J, Tommy D, Jacob Roland P, Henrik C, et al. Characterization of *Escherichia coli* causing cellulitis in broilers. Vet Microbiol. 2018;225: 72–78. doi: 10.1016/j.vetmic.2018.09.011
2018_Roschanski	AFEC	7	Roschanski N, Fischer J, Falgenhauer L, Pietsch M, Guenther S, Kreienbrock L, et al. Retrospective Analysis of Bacterial Cultures Sampled in German Chicken-Fattening Farms During the Years 2011-2012 Revealed Additional VIM-1 Carbapenemase-Producing *Escherichia coli* and a Serologically Rough *Salmonella enterica* Serovar Infantis. Front Microbiol. 2018;9.
2018_Wu	AFEC	64	Congming W, Yingchao W, Xiaomin S, Shuang W, Hongwei R, Zhangqi S, et al. Rapid rise of the ESBL and mcr-1 genes in *Escherichia coli* of chicken origin in China, 2008-2014. Emerg Microbes Infect. 2018;7: 30. Available: https://pubmed.ncbi.nlm.nih.gov/29535301/
2019_Abraham	AFEC	1	Sam A, Mark O, Shafi S, Kylie H, Anthony P, Tania V, et al. *Escherichia coli* and *Salmonella spp*. isolated from Australian meat chickens remain susceptible to critically important antimicrobial agents. PLoS ONE. 2019;14: e0224281. Available: https://pubmed.ncbi.nlm.nih.gov/31644602/
2019_Cummins	APEC	97	Cummins ML, Reid CJ, Chowdhury PR, Bushell RN, Esbert N, Tivendale KA, et al. Whole genome sequence analysis of Australian avian pathogenic *Escherichia coli* that carry the class 1 integrase gene. Microb Genomics. 2019;5. doi: 10.1099/mgen.0.000250
2019_Li	AFEC	2	Jiyun L, Zhenwang B, Shizhen M, Baoli C, Chang C, Junjia H, et al. Inter-host Transmission of Carbapenemase-Producing *Escherichia coli* among Humans and Backyard Animals. Environ Health Perspect. 2019;127: 107009. Available: https://pubmed.ncbi.nlm.nih.gov/31642700/
2019_Liu	AFEC	4	Liu ZY, Xiao X, Li Y, Liu Y, Li RC, Wang ZQ. Emergence of IncX3 Plasmid-Harboring bla(NDM)(-5) Dominated by *Escherichia coli* ST48 in a Goose Farm in Jiangsu, China. Front Microbiol. 2019;10.
2019_Maciuca	AFEC	94	Maciuca IE, Cummins ML, Cozma AP, Rimbu CM, Guguianu E, Panzaru C, et al. Genetic Features of mcr-1 Mediated Colistin Resistance in CMY-2-Producing *Escherichia coli* From Romanian Poultry. Front Microbiol. 2019;10: 2267. doi: 10.3389/fmicb.2019.02267
2019_Zajac	AFEC	77	Zajac M, Sztromwasser P, Bortolaia V, Leekitcharoenphon P, Cavaco LM, Zietek-Barszcz A, et al. Occurrence and Characterization of mcr-1-Positive *Escherichia coli* Isolated From Food-Producing Animals in Poland, 2011-2016. Front Microbiol. 2019;10.
2019_Zhuge	APEC	10	Xiangkai Z, Min J, Fang T, Yu S, Y Ji, Feng X, et al. Avian-source mcr-1-positive *Escherichia coli* is phylogenetically diverse and shares virulence characteristics with E. coli causing human extra-intestinal infections. Vet Microbiol. 2019;239: 108483. doi: 10.1016/j.vetmic.2019.108483
2020_Ahmed	AFEC	31	Ahmed S, Das T, Islam MZ, Herrero-Fresno A, Biswas PK, Olsen JE. High prevalence of mcr-1-encoded colistin resistance in commensal *Escherichia coli* from broiler chicken in Bangladesh. Sci Rep. 2020;10.
2020_Apostolakos	AFEC	60	Apostolakos I, Feudi C, Eichhorn I, Palmieri N, Fasolato L, Schwarz S, et al. High-resolution characterisation of ESBL/pAmpC-producing *Escherichia coli* isolated from the broiler production pyramid. Sci Rep. 2020;10. doi: 10.1038/s41598-020-68036-9
2020_Azam	APEC	55	Mariya A, Mashkoor M, Timothy J. J, Emily A. S, Abigail J, Muhammad U, et al. Genomic landscape of multi-drug resistant avian pathogenic *Escherichia coli* recovered from broilers. Vet Microbiol. 2020;247: 108766. doi: 10.1016/j.vetmic.2020.108766
2020_Flament-Simon	APEC	3	Flament-Simon SC, de Toro M, Chuprikova L, Blanco M, Moreno-González J, Salas M, et al. High diversity and variability of pipolins among a wide range of pathogenic *Escherichia coli* strains. Sci Rep. 2020;10. doi: 10.1038/s41598-020-69356-6
2020_Kaspersen	AFEC	90	Håkon K, Camilla S, Eve Zeyl F, Jannice Schau S, Roger S, Madelaine N, et al. Dissemination of Quinolone-Resistant *Escherichia coli* in the Norwegian Broiler and Pig Production Chains and Possible Persistence in the Broiler Production Environment. Appl Environ Microbiol. 2020;86. Available: https://pubmed.ncbi.nlm.nih.gov/31953334/
2020_Li	APEC	1	Li T, Castañeda CD, Arick MA, Hsu CY, Kiess AS, Zhang L, et al. Complete genome sequence of multidrug-resistant avian pathogenic *Escherichia coli* strain APEC-O2-MS1170. J Glob Antimicrob Resist. 2020;23: 401–403. doi: 10.1016/j.jgar.2020.11.009
2020_Mo	AFEC	31	Mo SS, Telke AA, Osei KO, Sekse C, Slettemeas JS, Urdahl AM, et al. bla(CTX-M-)(1)/IncI1-I gamma Plasmids Circulating in *Escherichia coli* From Norwegian Broiler Production Are Related, but Distinguishable. Front Microbiol. 2020;11.
2020_Papouskova	APEC	26	Papouskova A, Papouskova A, Masarikova M, Masarikova M, Valcek A, Valcek A, et al. Genomic analysis of *Escherichia coli* strains isolated from diseased chicken in the Czech Republic. BMC Vet Res. 2020;16. doi: 10.1186/s12917-020-02407-2
2020_Poulsen	APEC	62	Poulsen LL, Kudirkiene E, Jørgensen SL, Djordjevic SP, Cummins ML, Christensen JP, et al. Whole genome sequence comparison of avian pathogenic *Escherichia coli* from acute and chronic salpingitis of egg laying hens. BMC Vet Res. 2020;16: 1–9. doi: 10.1186/s12917-020-02369-5
2020_Rafique	APEC	92	Rafique M, Potter RF, Ferreiro A, Wallace MA, Rahim A, Malik AA, et al. Genomic Characterization of Antibiotic Resistant *Escherichia coli* Isolated From Domestic Chickens in Pakistan. Front Microbiol. 2020;10.
2021_Apostolakos	APEC	23	Apostolakos I, Laconi A, Mughini-Gras L, Yapicier ÖS, Piccirillo A. Occurrence of Colibacillosis in Broilers and Its Relationship With Avian Pathogenic *Escherichia coli* (APEC) Population Structure and Molecular Characteristics. Front Vet Sci. 2021;8: 1040. doi: 10.3389/fvets.2021.737720
2021_Chen	APEC	51	Chen X, Liu W, Li H, Yan S, Jiang F, Cai W, et al. Whole genome sequencing analysis of avian pathogenic *Escherichia coli* from China. Vet Microbiol. 2021;259. doi: 10.1016/j.vetmic.2021.109158
2021_Ewers	AFEC	45	Ewers C, de Jong A, Prenger-Berninghoff E, El Garch F, Leidner U, Tiwari SK, et al. Genomic Diversity and Virulence Potential of ESBL- and AmpC-β-Lactamase-Producing *Escherichia coli* Strains From Healthy Food Animals Across Europe. Front Microbiol. 2021;12. doi: 10.3389/fmicb.2021.626774
2021_Lozica	APEC	112	Lozica L, Repar J, Gottstein Ž. Longitudinal study on the effect of autogenous vaccine application on the sequence type and virulence profiles of *Escherichia coli* in broiler breeder flocks. Vet Microbiol. 2021;259: 109159. doi: 10.1016/j.vetmic.2021.109159
2021_Mageiros	AFEC+APEC	476	Mageiros L, Méric G, Bayliss SC, Pensar J, Pascoe B, Mourkas E, et al. Genome evolution and the emergence of pathogenicity in avian *Escherichia coli*. Nat Commun. 2021;12. doi: 10.1038/s41467-021-20988-w
2021_Wang	APEC	54	Wang Z, Zheng X, Guo G, Dong Y, Xu Z, Wei X, et al. Combining pangenome analysis to identify potential cross-protective antigens against avian pathogenic *Escherichia coli*. Avian Pathol. 2022;51: 66–75. doi: 10.1080/03079457.2021.2005240
2021_Yin	APEC	3	Yin D, Cheng B, Yang K, Xue M, Lin Y, Li Z, et al. Complete Genetic Analysis of Plasmids Carrying mcr-1 and Other Resistance Genes in Avian Pathogenic *Escherichia coli* Isolates from Diseased Chickens in Anhui Province in China. mSphere. 2021;6. doi: 10.1128/msphere.01135-20

Columns from left to right include: publication (Year_FirstAuthor), pathotype of strains analyzed in that publication, number of isolates analyzed and full citation.

### Summary of the avian *E. coli* genomic database

**Figure 1:**
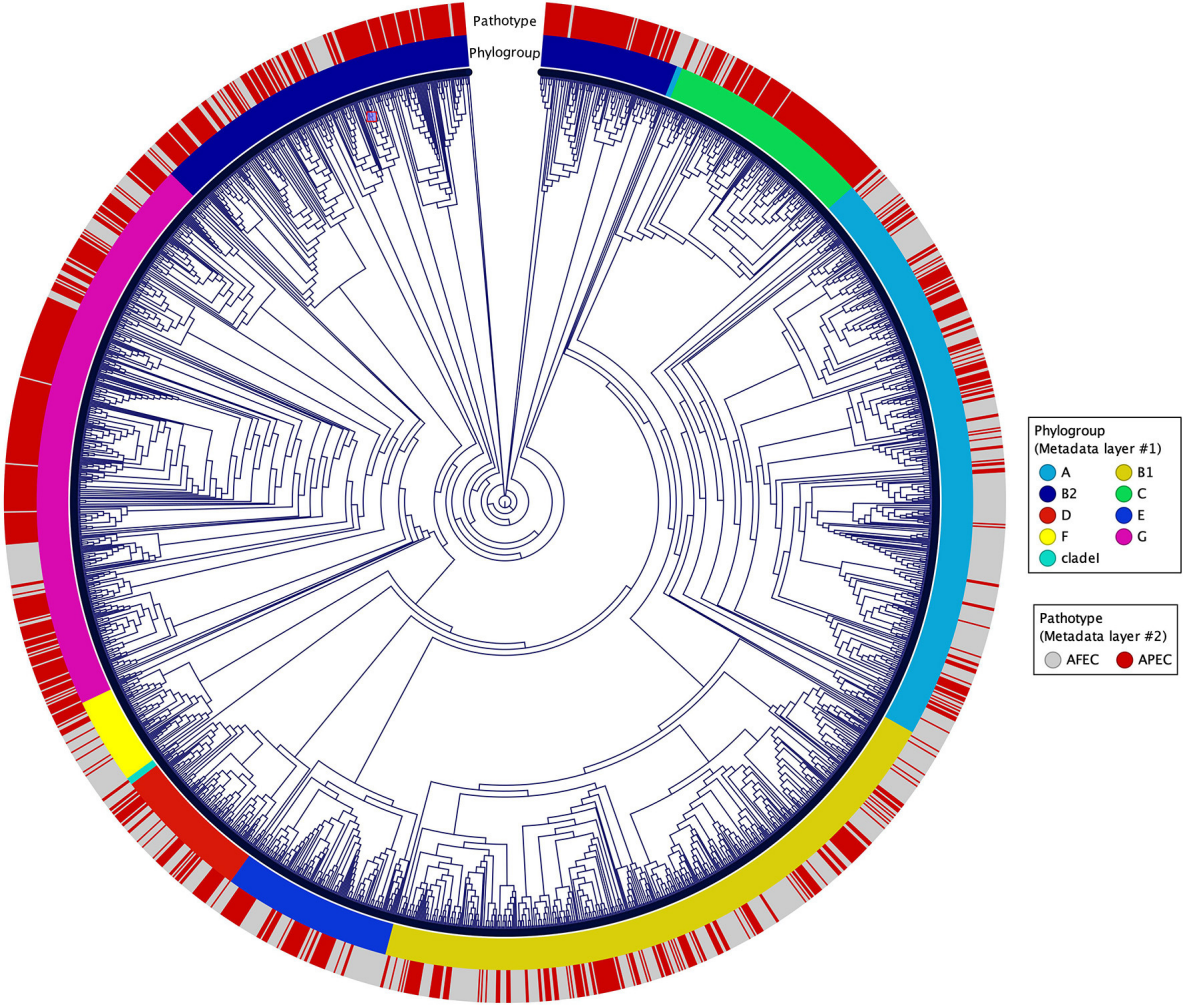
Circular cladogram for the 2015 avian *E. coli* isolates. The circular cladogram shows the relationships between the 2015 avian *E. coli* isolates, with the outermost circle indicating the pathotypes, and the second circle indicating the phylogroups highlighted in different colors.

**Figure 2:**
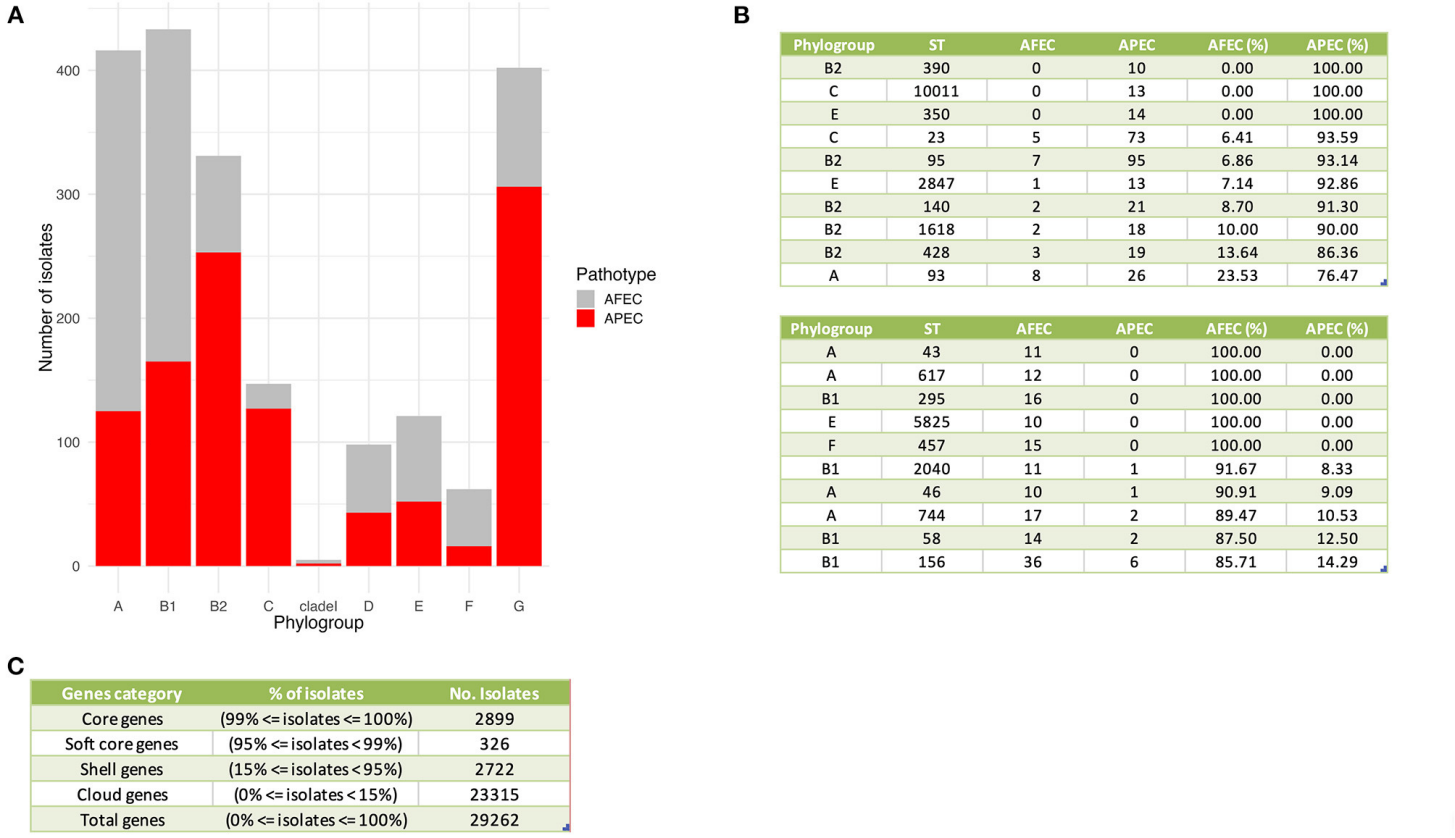
General genomic features of APEC and AFEC isolates. **(A)** Distribution of APEC and AFEC isolates by phylogroup. **(B)** Top 10 sequence types with at least 10 isolates associated with APEC isolates (top table) and AFEC isolates (bottom table). Columns from left to right indicate: phylogroup, sequence type (ST), number of AFEC isolates, number of APEC isolates, and the percentage of each group with that ST. **(C)** Division of genes in the pan-genome of 2015 *E. coli* isolates based on their prevalence among the isolates. The genes are categorized as core, soft core, shell, or cloud genes, depending on the percentage of isolates in which they are present. The table displays the percentage of isolates and the number of isolates for each gene category, as well as the total number of genes in the pan-genome.

A K-mer tree in form of a circular cladogram for the curated 2015 avian *E. coli* isolates is depicted in Figure 1 highlighting the connection of pathotype with phylogroups in different colors. Isolates from each pathotype were unequally distributed in each phylogroup (Chi-Square test – P = 1.04 × 10^−77^) (Figure 2A), with phylogroups B2, C, and G significantly enriched in APEC isolates (Chi-Square *post-hoc* test – P_B2_ = 0, P_C_ = 0 and P_G_ = 0) and phylogroups A and F significantly enriched in AFEC isolates (Chi-Square *post-hoc* test – P_A_ = 0 and P_F_ = 0.0001). Genome length distribution is significantly different between APEC and AFEC isolates (Mann-Whitney U test – P = 0.0225), with APEC genomes being significantly shorter than AFEC genomes (median length _APEC_ = 5.127 Mbp, median length _AFEC_ = 5.142 Mbp). However, when splitting the data by phylogroup, APEC genomes were significantly shorter than AFEC genomes for phylogroups B1, E and G only (Mann-Whitney U test – P_B1_ = 8.99 × 10^−12^, P_E_ = 8.16 × 10^−6^, P_G_ = 9.09 × 10^−8^), indicating that shorter genome length is not an exclusive feature of APEC isolates. Gene number distribution is also significantly different between APEC and AFEC isolates (Mann-Whitney U test – P = 0.0095), with APEC isolates having significantly less genes than AFEC isolates (median no. genes _APEC_ = 4888, median no. genes _AFEC_ = 4916). When splitting the data by phylogroup, this pattern holds only for phylogroups B1, E, G (Mann-Whitney U test – P_B1_ = 8.23 × 10^−11^, P_E_ = 1.25 × 10^−5^, P_G_ = 3.79 × 10^−8^) implying that a lower number of genes might simply reflect a shorter genome length and is not a general feature of APEC isolates. In Figure 2B we report the top 10 sequence types with at least 10 isolates, sorted by prevalence in APEC isolates (top table) and AFEC isolates (bottom table), which provides additional insights into the genetic diversity of the isolates. The pan-genome of the 2015 *E. coli* isolates consisted of 29,262 genes, which were divided into 2,899 core and 26,363 accessory genes (Figure 2C).

### Contribution of different genomic variations to APEC isolates

**Figure 3:**
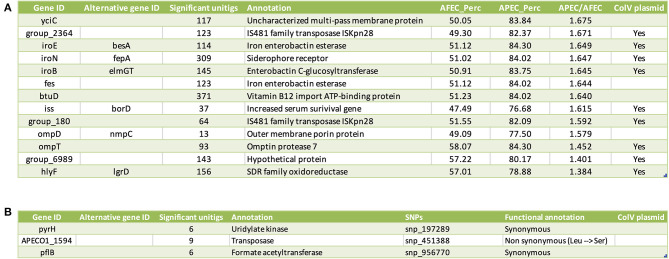
List of candidate genes linked to the APEC pathotype. **(A)** Candidate genes identified through the gene-based GWAS. Columns from left to right indicate: gene ID, alternative gene ID(s), functional annotation of the gene, prevalence of the gene (in %) among AFEC isolates (AFEC_Perc), prevalence of the gene (in %) among APEC isolates (APEC_Perc), and the prevalence ratio of APEC_Perc over AFEC_Perc. Additionally, a column indicates whether the gene is located on the ColV plasmid. **(B)** Candidate genes identified through the SNP-based GWAS. columns from left to right indicate: gene ID, alternative gene ID(s), functional annotation of the gene, and significant SNPs, which are encoded as snp_xxxxxx_y. Here, xxxxxx represents the position of the SNP in the genome whereas y stands for a distinct numerical value (1, 2, 3) that uniquely distinguishes each alternative allele.

To assess the relative contributions of gene-level and SNP-level genomic variation associated with APEC isolates, we initially performed two independent GWAS: (1) a gene-based GWAS to identify associations between the phenotypic vector of each strain (1 = APEC, 0 = AFEC) and variation in gene content defined by a gene presence/absence matrix; (2) an SNP-based GWAS to find associations between the phenotypic vector of each strain (1 = APEC, 0 = AFEC) and an SNPs matrix derived from the whole-genome alignment of our 2015 isolates against the APEC O1 reference strain. Out of a total of 29,262 genes in the pan-genome, the gene-based GWAS identified 16 genes significantly associated with APEC isolates. From a total of 22,920 SNPs in the core genome, the SNP-based GWAS found 7 SNPs significantly associated with APEC isolates, located in 6 different genes. To further validate these candidate genes, we employed a unitig-based GWAS. This approach validated 13 of the 16 genes from the gene-based GWAS (Figure 3A) and 3 of the 6 genes from the SNP-based GWAS (Figure 3B). In total, we identified 16 final candidate genes linked to APEC isolates after considering the results from both gene-based and SNP-based GWAS, along with the unitig-based validation.

### Characterization of genes linked to APEC isolates

**Figure 4:**
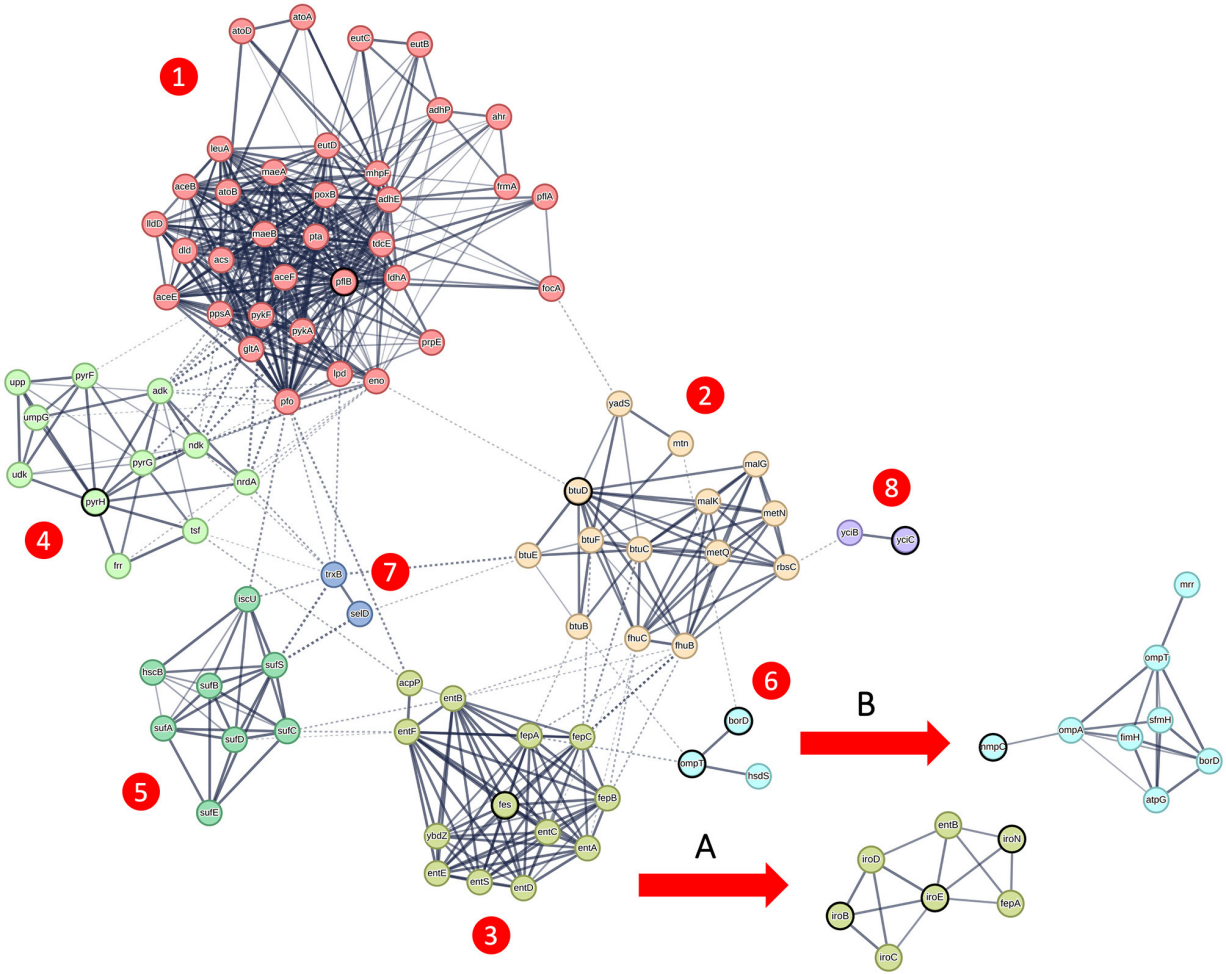
Genetic network of candidate genes linked to the APEC pathotype. Genetic network obtained from the STRING database (22), featuring candidate genes emphasized in bold. The core genetic network, originating from strain K12 MG1655, displays genes color-coded based on MCL clustering using an inflation parameter of 3. These genes are organized into seven distinct clusters, numbered 1 through 7. Additionally, subnetwork A, indicated by the arrow, is derived from strain CFT073, while subnetwork B originates from strain K12 MG1655.

To further investigate the 16 candidate genes associated with APEC isolates, we utilized protein–protein interaction networks from the STRING database (22). As these data are only available for genes with standard 3 or 4 letter gene names, we excluded the 4 candidate genes with generic gene names (group_2364, group_180, group_6989, and APECO1_1594) from this analysis. Among the remaining 12 candidate genes, protein–protein interaction data were identified for 11 of them (all of them except *hlyF*). These included 7 genes that belonged to the primary genetic network (Figure 4—main network) derived from strain K12 MG1655, 3 genes related to subnetwork A (Figure 4) derived from strain CFT073, and 1 gene linked to subnetwork B (Figure 4) derived from strain UMN026. It is important to note that subnetwork B shares two genes (*ompT* and *borD*) in common with the primary genetic network; however, they are not circled in bold in subnetwork B to avoid confusion, as they are already highlighted in the primary network. Altogether, these interactions encompassed a total of 99 genes. In order to unambiguously describe this network, we applied MCL clustering with inflation parameter = 3, colored each node based on the membership to the respective cluster, and numbered the clusters from 1 to 8. To avoid ambiguities, we will report the alternative gene names throughout the manuscript in case of genes with multiple gene names.

Cluster 1 includes the gene formate acetyltransferase (*pflB*) and is linked to pyruvate metabolism (*P* = 2.43e-17), acetate metabolic process and ethanol biosynthetic process (*P* = 4.68e-05). Cluster 2 includes the vitamin B12 import ATP-binding protein (*btuD*) and is linked to cobalamin transport (*P* = 2.34e-05), nitrogen compound transport (*P* = 0.0043) and organic substance transport (*P* = 0.00019).

Cluster 3 together with subnetwork A includes the genes iron enterobactin esterase (*fes*) enterobactin C-glucosyltransferase (*iroB*), iron enterobactin esterase (*iroE*) and siderophore receptor (*iroN*) and is enriched for enterobactin metabolic process (*P* = 3.35e-14), siderophore-dependent iron import in the cell (*P* = 1.38e-05) and colicin transport (*P* = 0.0034). Cluster 4 includes the gene (*pyrH*) and is related to CTP metabolic process (*P* = 8.50e-07) and ribonucleoside monophosphate biosynthetic process (*P* = 2.11e-06). Cluster 5 does not contain any candidate gene but it connects clusters 1 and 3. This cluster is enriched for sulfur incorporation into metallo-sulfur cluster (*P* = 0.0042) and iron-sulfur cluster assembly.

Cluster 6 together with subnetwork B includes the genes omptin protease (*ompT*), increased serum survival gene (*borD* – more commonly known as iss), and outer membrane porin (*nmpC*, also known as *ompD*) and is linked to restriction system, and Bor protein (*P* = 2.45e-05) and signal peptide (*P* = 0.0040). Cluster 7 do not contain candidate genes and it is connected to selenocompound metabolism (*P* = 0.0023). Cluster 8 contains the uncharacterized multi-pass membrane protein (*yciC*) and is linked to intracellular septation protein A and uncharacterized protein family (*P* = 0.0033).

The remaining candidate gene with standard gene names without protein–protein interaction data encodes for a SDR family oxidoreductase (*hlyF*). Further characterization using the KEGG database did not provide additional functional details for this gene. However, it is known that this gene encodes for a virulence factor involved in outer membrane vesicle biogenesis in ExPEC strains (5).

Finally, the 4 candidate genes with generic names encode for three transposase genes (*group_2364, group_180, APECO1_1594*) and for a hypothetical protein (*group_6989*). For these genes, we looked at their genomic location on the APEC O1 reference strain and observed that 3 out of 4 are located on the ColV plasmid. More in detail, the IS418 family transposases *group_2364* and *group_180* are located in the region upstream to the candidate gene *iss* (Supplementary Figure 2A); while the hypothetical protein *group_6989* is adjacent to the candidate gene *hlyF* (Supplementary Figure 2B).

## Discussion

In an effort to elucidate the underlying genetic network of avian *E. coli* involved in colibacillosis, we collected all available genomes of relevant isolates reported in the period 2000-2021 and constructed a comprehensive dataset including 2015 avian *E. coli* isolates covering a wide range of APEC and AFEC strains. Our aim was to construct a sufficiently large database in order to conduct a robust GWAS analysis (23).

Two major trends emerged from the exploratory analysis of this dataset. First, we found that APEC strains can emerge from any phylogroup; however, phylogroups B2, C, and G were significantly enriched with APEC strains, consistent with the results from Johnson et al. (8). Second, we observed that APEC genomes are significantly shorter than AFEC genomes. This is in line with the observation that many pathogenic bacteria have usually smaller genomes compared to their non-pathogenic counterpart (24). This might be an effect of host adaptation, for which some genes could either become less necessary for survival or could be selectively lost if they encode for immune targets. This finding adds another unexplored layer in explaining the evolution of APEC pathogenicity.

Our GWAS analysis identified variation in gene content for 13 genes and SNPs in 3 different genes as linked to APEC isolates. Thus, our findings suggest that both variations at gene-level and SNP-level contribute to the APEC pathotype. By integrating available protein–protein interaction data for these 16 genes, we showed that 15 of them clustered in the same genetic network connecting a total of 99 genes. This result suggests that APEC pathogenicity might be the result of the interplay of different tightly regulated pathways.

Based on the results from our genetic network analysis, we classified the candidate genes into four categories: (1) genes associated with nutrient uptake; (2) genes involved in defense against the host immune system; (3) genes co-selected due to their proximity to other candidate genes; and (4) previously unidentified genes that are now linked to APEC isolates.

In the first category of candidate genes, iron uptake seems to play a major role in APEC pathogenicity. The role of iron uptake in APEC pathogenicity is well documented (25): the genes from the *iroBCDEN* operon can be located on the ColV plasmid (9) or chromosomally encoded and are involved in glycosylation (*iroB*), processing (*iroE*) and transport (*iroN*) of the salmocheline siderophore (26), a protein able to sequester iron from host cells. These proteins were functionally investigated in the APEC O78 strain and found to be essential for APEC pathogenicity (26). The *fes* gene is also involved in biosynthesis of salmocheline and its deletion in mutants of the APEC strain χ7122 caused a reduction of salmocheline production (27), but not a complete ablation of the product. The role of iron uptake was also confirmed in a recent GWAS study on ExPEC strains (28).

The genetic network further suggests that the uptake of other nutrients might be also important for APEC pathogenicity: these include vitamin B12 uptake, connected to the vitamin B12 import ATP-binding protein (*btuD*) and acetate assimilation, linked to the formate acetyltransferase (*pflB*) gene. While the role of vitamin B12 in APEC pathogenicity was never reported before, a study performed on two highly virulent APEC strains highlighted that the deletion of three genes directly connected to the *pflB* gene (*acs, yjcH* and *actP*) caused a decrease of cytotoxicity in macrophages, which was linked to the ablation of the acetate assimilation system (29). Thus, the authors concluded that acetate assimilation conferred a fitness advantage during APEC early colonization. Based on these results, it appears that ability to uptake iron, vitamin B12 and acetate from the host promotes the emergence of pathogenicity in avian *E. coli*.

The second category of candidate genes includes genes involved in the defense from host-immune system, among which the *ompT, borD* (*iss*), and *hlyF* genes. The omptin protease (*ompT*) is located on the ColV plasmid (9) and is involved in the degradation of host proteins and antimicrobial peptides (30). It was reported as a standard APEC marker gene (7) and its role in APEC pathogenicity has been confirmed in a genetic deletion study in the APEC strain TW-XM, where it affected adhesion, invasion, colonization, and proliferation capacities (31). Closely linked to *ompT* in the genetic network, lays the *borD* gene, more commonly known as increased serum survival gene (*iss*), also located on the ColV plasmid (9) and expressed on the outer membrane (32). The iss has been also extensively used as APEC marker (7) and a deletion in the APEC O78-9 strain affected its ability to grow in serum (33), but did not provide a detailed functional characterization. Only recently, a functional study in ExPEC strains revealed its role in group 4 capsule synthesis (34), which protects the bacteria from the complement proteins of the host immune system. The SDR family oxidoreductase (*hlyF*) gene is also located on the ColV plasmid and one of the standard APEC markers (32). This gene was originally thought to be a hemolysin, however a functional study showed that it is involved in the regulation of outer membrane vesicle biogenesis (35), which prevents the autophagosome-lysosome fusion during the immune response, amplifying the pathogenic potential of strains containing this gene (36). These results suggest that a combination of host-specific immune-defense mechanisms contribute to the appearance of pathogenicity in avian *E. coli*.

The third category of candidate genes includes genes that might be co-selected due to proximity to other candidate genes. These include: (1) two transposase genes (*group_2364, group_180*), which could be involved in horizontal gene transfer of other candidate genes; (2) the hypothetical protein (*group_6989*), which might be simply co-transposed together with *hlyF* due to their adjacent position.

In addition to already known genes, we also revealed three previously unidentified genes associated with APEC isolates: (1) an uncharacterized multi-pass membrane protein (*yciC*), encoding for a hypothetical protein containing six transmembrane domains; remarkably, this gene had the highest APEC/AFEC prevalence ratio in the gene-based GWAS analysis, pointing to a potential but unknown pivotal role in APEC pathogenicity; (2) the outer membrane porin (*nmpC*, more commonly known as *ompD*), that was previously found to be overexpressed in a mutant of the APEC strain DE17 deleted for the *pfs* gene (37), suggesting a possible role in antibiotic resistance; (3) a significant SNP in the *pyrH* gene, which is a synonymous mutation. This gene encodes uridylate kinase (UMPK), an enzyme involved in pyrimidine nucleotide metabolism. Since this mutation does not impact the protein sequence, we initially hypothesized that the association of this SNP with APEC isolates could potentially be explained by genetic hitchhiking, where the mutation's frequency in the population increases due to its close linkage to another candidate gene linked to APEC. However, upon screening the chromosomal locations of candidate genes in the APEC O1 strain, we found that none of them were in close proximity to *pyrH*, making this hypothesis less likely. Alternatively, the linkage of this SNP to APEC isolates might be explained by genetic epistasis: the mutation in *pyrH* could interact with other genetic variants elsewhere in the genome, leading to a combined effect that results in an association with APEC isolates.

Two other GWAS analyses were previously published to address the question of finding genes linked to the APEC pathotype. The first GWAS, by Mageiros et al. (5), was performed on 568 isolates, which are also included in our dataset. Their GWAS analysis identified 143 genes involved in metabolism, lipopolysaccharide synthesis, heat shock response, antimicrobial resistance and toxicity. From the subset of 10 candidate genes with standard names from our gene-based GWAS, only 1 (*hlyF*) was also detected by Mageiros et al. (5). This may be explained by their approach to conduct the analysis separately for each phylogroup, thus drastically reducing GWAS statistical power. The second GWAS, by Johnson et al. (8), was published after we terminated our data collection phase, and was conducted on 959 clinical and caecal isolates from turkeys, which are part of a larger dataset including a total of 3,479 isolates. Their estimate of 430 candidate genes is higher compared to our 13 genes from the gene-based GWAS. This may be explained by the less conservative filtering criteria applied by Johnson et al. (8). Remarkably, 8 of the 10 candidate genes with standard names from our gene-based GWAS were also found by Johnson et al. (*yciC, iroE, iroN, iroB, btuD, iss, ompT, hlyF*), providing independent validation for our findings. This overlap further confirms the hypothesis that the genes underlying APEC pathogenesis are common between chicken and turkey, as corroborated by Johnson's GWAS conducted specifically on turkey strains. Interestingly, this list includes the uncharacterized multi-pass membrane protein (*yciC*), which we proposed as an attractive novel candidate for functional testing. This discovery suggests the importance of investigating the potential role of *yciC* in APEC pathogenesis and its potential as a therapeutic target for controlling APEC infections in poultry. In conclusion, our results suggest that the interplay between nutrient uptake and the ability to escape host immune defenses enable the emergence of pathogenicity in avian extra-intestinal *E. coli*.

## Data availability statement

The original contributions presented in the study are included in the article/Supplementary material, further inquiries can be directed to the corresponding author.

## Author contributions

NP and IA conceived, designed the study, and performed the data analysis. SP and MH contributed to data interpretation and provided critical insights. All authors participated in drafting the manuscript, revising it for important intellectual content, and provided final approval for publication.

## Supplementary material

Supplementary Figure 1 Pipeline for the identification of genes linked to the APEC pathotype. Genome assemblies were downloaded from NCBI (unfiltered genomes), and reads data were assembled using the PATRIC web application if the genome sequences were not available. The quality of the assemblies was assessed using checkM, and low-quality genomes were filtered out based on several criteria (filtered genomes). The genomes were annotated using PROKKA (Genes annotation) and a Maximum Likelihood tree was constructed with FastTree. For the gene-based GWAS, a gene presence/absence matrix was produced with Panaroo, and treeWAS was used to identify significant genes associated with APEC isolates. Candidate genes with a prevalence > 50% in APEC and APEC vs. AFEC prevalence ratio > 1 were selected. For the SNP-based GWAS, parsnp was used to align the strains and generate a VCF file of SNPs, which were filtered and processed using bcftools. Significant SNPs associated with APEC isolates were identified using treeWAS. A unitig-based GWAS was conducted to further validate the significant genes identified from gene-based and SNP-based GWAS. The unitigs were generated and assessed for association with APEC phenotype, and the significant unitigs were aligned to the candidate genes using BLASTN. Final candidates were selected by retaining only the genes with at least 5 aligned unitigs.

Supplementary Figure 2 Genomic locations of candidate genes with generic gene names. Each panel contains two tracks from top to bottom: genes and candidate genes. **(A)** Locations of *group_2364* and *group_180* on the ColV plasmid from the APEC O1 strain with respect to other candidate genes; **(B)** Locations of *group_6989* on the ColV plasmid from the APEC O1 strain with respect to other candidate genes.

Supplementary Table 1 Metadata for the 2015 avian *E. coli* isolates. Columns from left to right indicate: name of the isolate, publication (Year_FirstAuthor), host, pathotype (APEC/AFEC), genome length (bp), number of genes, phylogroup, sequence type (ST), name of the allele variant for the 7 genes used to define the sequence type by the ClermontTyping tool (15).

## References

[1] Nolan LK John BH Vaillancourt JP Abdul-Aziz T Logue CM. Colibacillosis. Diseases of Poultry. 13th ed. Chichester: John Wiley & Sons, Ltd. (2017). p. 751–805.

[2] Kaufmann-Bat M Hoop RK. Diseases in chicks and laying hens during the first 12 years after battery cages were banned in Switzerland. Vet Record. (2009) 164:203–7. 10.1136/vr.164.7.203 19218590

[3] Grafl B Gaußmann B Sulejmanovic T Hess C Hess M. Risks and disease aetiologies of compromised performance in commercial broilers kept at lower stocking density and limited antimicrobial use. Avian Pathol. (2020) 49:621–30. 10.1080/03079457.2020.1805411 32746625

[4] Optimising Poultry Flock Health | Sjaak de Wit | Taylor & Francis eBoo. Available online at: https://www.taylorfrancis.com/pdfviewer/ (accessed September 26, 2022).

[5] Mageiros L Méric G Bayliss SC Pensar J Pascoe B Mourkas E. Genome evolution and the emergence of pathogenicity in avian *Escherichia coli*. Nat Commun. (2021) 12:765. 10.1038/s41467-021-20988-w 33536414 PMC7858641

[6] Ewers C Janßen T Kießling S Philipp HC Wieler LH. Rapid detection of virulence-associated genes in avian pathogenic *Escherichia coli* by multiplex polymerase chain reaction. Avian Dis. (2005) 49:269–73. 10.1637/7293-102604R 16094833

[7] Johnson TJ Wannemuehler Y Doetkott C Johnson SJ Rosenberger SC Nolan LK. Identification of minimal predictors of avian pathogenic *Escherichia coli* virulence for use as a rapid diagnostic tool. J Clin Microbiol. (2008) 46:3987–96. 10.1128/JCM.00816-08 18842938 PMC2593276

[8] Johnson TJ Miller EA Flores-Figueroa C Munoz-Aguayo J Cardona C Fransen K. Refining the definition of the avian pathogenic *Escherichia coli* (APEC) pathotype through inclusion of high-risk clonal groups. Poult Sci. (2022) 101:102009. 10.1016/j.psj.2022.102009 35952599 PMC9385700

[9] Johnson TJ Siek KE Johnson SJ Nolan LK. DNA sequence of a ColV plasmid and prevalence of selected plasmid-encoded virulence genes among avian *Escherichia coli* strains. J Bacteriol. (2006) 188:745–58. 10.1128/JB.188.2.745-758.2006 16385064 PMC1347294

[10] Saber MM Jesse Shapiro B. Benchmarking bacterial genome-wide association study methods using simulated genomes and phenotypes. Microb Genom. (2020) 6:e000337. 10.1099/mgen.0.000337 32100713 PMC7200059

[11] Ouzzani M Hammady H Fedorowicz Z Elmagarmid A. Rayyan-a web and mobile app for systematic reviews. Syst Rev. (2016) 5:1. 10.1186/s13643-016-0384-4 27919275 PMC5139140

[12] Davis JJ Wattam AR Aziz RK Brettin T Butler R Butler RM. The PATRIC bioinformatics resource center: expanding data and analysis capabilities. Nucleic Acids Res. (2020) 48:D606–D612. 10.1093/nar/gkz943 31667520 PMC7145515

[13] Parks DH Imelfort M Skennerton CT Hugenholtz P Tyson GW. CheckM: assessing the quality of microbial genomes recovered from isolates, single cells, and metagenomes. Genome Res. (2015) 25:1043–55. 10.1101/gr.186072.114 25977477 PMC4484387

[14] Seemann T. Prokka: rapid prokaryotic genome annotation. Bioinformatics. (2014) 30:2068–9. 10.1093/bioinformatics/btu153 24642063

[15] Beghain J Bridier-Nahmias A Nagard H le Denamur E Clermont O. ClermonTyping: an easy-to-use and accurate in silico method for Escherichia genus strain phylotyping. Microb Genom. (2018) 4:e000192. 10.1099/mgen.0.000192 29916797 PMC6113867

[16] Tonkin-Hill G MacAlasdair N Ruis C Weimann A Horesh G Lees JA. Producing polished prokaryotic pangenomes with the Panaroo pipeline. Genome Biol. (2020) 21:1–21. 10.1186/s13059-020-02090-4 32698896 PMC7376924

[17] Price MN Dehal PS Arkin AP. Fasttree: computing large minimum evolution trees with profiles instead of a distance matrix. Mol Biol Evol. (2009) 26:1641–50. 10.1093/molbev/msp077 19377059 PMC2693737

[18] Collins C Didelot X. A phylogenetic method to perform genome-wide association studies in microbes that accounts for population structure and recombination. PLoS Comput Biol. (2018) 14:e1005958. 10.1371/journal.pcbi.1005958 29401456 PMC5814097

[19] Treangen TJ Ondov BD Koren S Phillippy AM. The harvest suite for rapid core-genome alignment and visualization of thousands of intraspecific microbial genomes. Genome Biol. (2014) 15:1–15. 10.1186/s13059-014-0524-x 25410596 PMC4262987

[20] Danecek P Bonfield JK Liddle J Marshall J Ohan V Pollard MO. Twelve years of SAMtools and BCFtools. Gigascience. (2021) 10:giab008. 10.1093/gigascience/giab008 33590861 PMC7931819

[21] Saund K Lapp Z Thiede SN Pirani A Snitkin ES. Prewas: data pre-processing for more informative bacterial gwas. Microb Genom. (2020) 6:1–8. 10.1099/mgen.0.000368 32310745 PMC7371116

[22] Szklarczyk D Gable AL Nastou KC Lyon D Kirsch R Pyysalo S. The STRING database in 2021: Customizable protein-protein networks, and functional characterization of user-uploaded gene/measurement sets. Nucleic Acids Res. (2021) 49:D605–12. 10.1093/nar/gkab835 33237311 PMC7779004

[23] Power RA Parkhill J de Oliveira T. Microbial genome-wide association studies: lessons from human GWAS. Nat Rev Genet. (2016) 18:41–50. 10.1038/nrg.2016.132 27840430

[24] Weinert LA Welch JJ. Why might bacterial pathogens have small genomes? Trends Ecol Evol. (2017) 32:936–47. 10.1016/j.tree.2017.09.006 29054300

[25] Gao Q Wang X Xu H Xu Y Ling J Zhang D. Roles of iron acquisition systems in virulence of extraintestinal pathogenic *Escherichia coli*: Salmochelin and aerobactin contribute more to virulence than heme in a chicken infection model. BMC Microbiol. (2012) 12:1–2. 10.1186/1471-2180-12-143 22817680 PMC3496646

[26] Caza M Lépine F Milot S Dozois CM. Specific roles of the iroBCDEN genes in virulence of an avian pathogenic *Escherichia coli* O78 strain and in production of salmochelins. Infect Immun. (2008) 76:3539–49. 10.1128/IAI.00455-08 18541653 PMC2493193

[27] Caza M Garénaux A Lépine F Dozois CM. Catecholate siderophore esterases Fes, IroD and IroE are required for salmochelins secretion following utilization, but only IroD contributes to virulence of extra-intestinal pathogenic *Escherichia coli*. Mol Microbiol. (2015) 97:717–32. 10.1111/mmi.13059 25982934

[28] Galardini M Clermont O Baron A Busby B Dion S Schubert S. Major role of iron uptake systems in the intrinsic extra-intestinal virulence of the genus Escherichia revealed by a genome-wide association study. PLoS Genet. (2020) 16:e1009065. 10.1371/journal.pgen.1009065 33112851 PMC7592755

[29] Zhuge X Sun Y Jiang M Wang J Tang F Xue F. Acetate metabolic requirement of avian pathogenic *Escherichia coli* promotes its intracellular proliferation within macrophage. Vet Res. (2019) 50:1–8. 10.1186/s13567-019-0650-2 31046828 PMC6498577

[30] Kukkonen M Korhonen TK. The omptin family of enterobacterial surface proteases/adhesins: from housekeeping in *Escherichia coli* to systemic spread of Yersinia pestis. Int J Med Microbiol. (2004) 294:7–14. 10.1016/j.ijmm.2004.01.003 15293449

[31] Hejair HMA Ma J Zhu Y Sun M Dong W Zhang Y. Role of outer membrane protein T in pathogenicity of avian pathogenic *Escherichia coli*. Res Vet Sci. (2017) 115:109–16. 10.1016/j.rvsc.2017.01.026 28199899

[32] Lynne AM Skyberg JA Logue CM Nolan LK. Detection of Iss and Bor on the surface of *Escherichia coli*. J Appl Microbiol. (2007) 102:660–6. 10.1111/j.1365-2672.2006.03133.x 17309614

[33] Huja S Oren Y Trost E Brzuszkiewicz E Biran D Blom J. Genomic avenue to avian colisepticemia. mBio. (2015) 6:e01681–14. 10.1128/mBio.01681-14 25587010 PMC4313913

[34] Biran D Sura T Otto A Yair Y Becher D Ron EZ. Surviving serum: the *Escherichia coli* is *gene of* extraintestinal pathogenic *E. coli* is required for the synthesis of group 4 capsule. Infect Immunity. (2021) 89:e00316–21. 10.1128/IAI.00316-21 34181459 PMC8445191

[35] Murase K Martin P Porcheron G Houle S Helloin E Pénary M. HlyF produced by extraintestinal pathogenic *Escherichia coli* is a virulence factor that regulates outer membrane vesicle biogenesis. J Infect Dis. (2015) 212:856–65. 10.1093/infdis/jiv506 26494774

[36] David L Taieb F Pénary M Bordignon PJ Planès R Bagayoko S. Outer membrane vesicles produced by pathogenic strains of *Escherichia coli* block autophagic flux and exacerbate inflammasome activation. Autophagy. (2022) 18:2913–25. 10.1080/15548627.2022.2054040 35311462 PMC9673956

[37] Hu J Che C Jiang W Chen Z Tu J Han X. Avian pathogenic *Escherichia coli* through Pfs affects the tran-scription of membrane proteins to resist β-lactam antibiotics. Vet Sci. (2022) 9:98. 10.3390/vetsci9030098 35324826 PMC8951488

