# Supplementary figures and images for: The genetic network underlying the evolution of pathogenicity in avian Escherichia coli
Source: Front Vet Sci. 2023 Jun 21;10:1195585. doi: 10.3389/fvets.2023.1195585 (PMC10321414; doi:10.3389/fvets.2023.1195585)

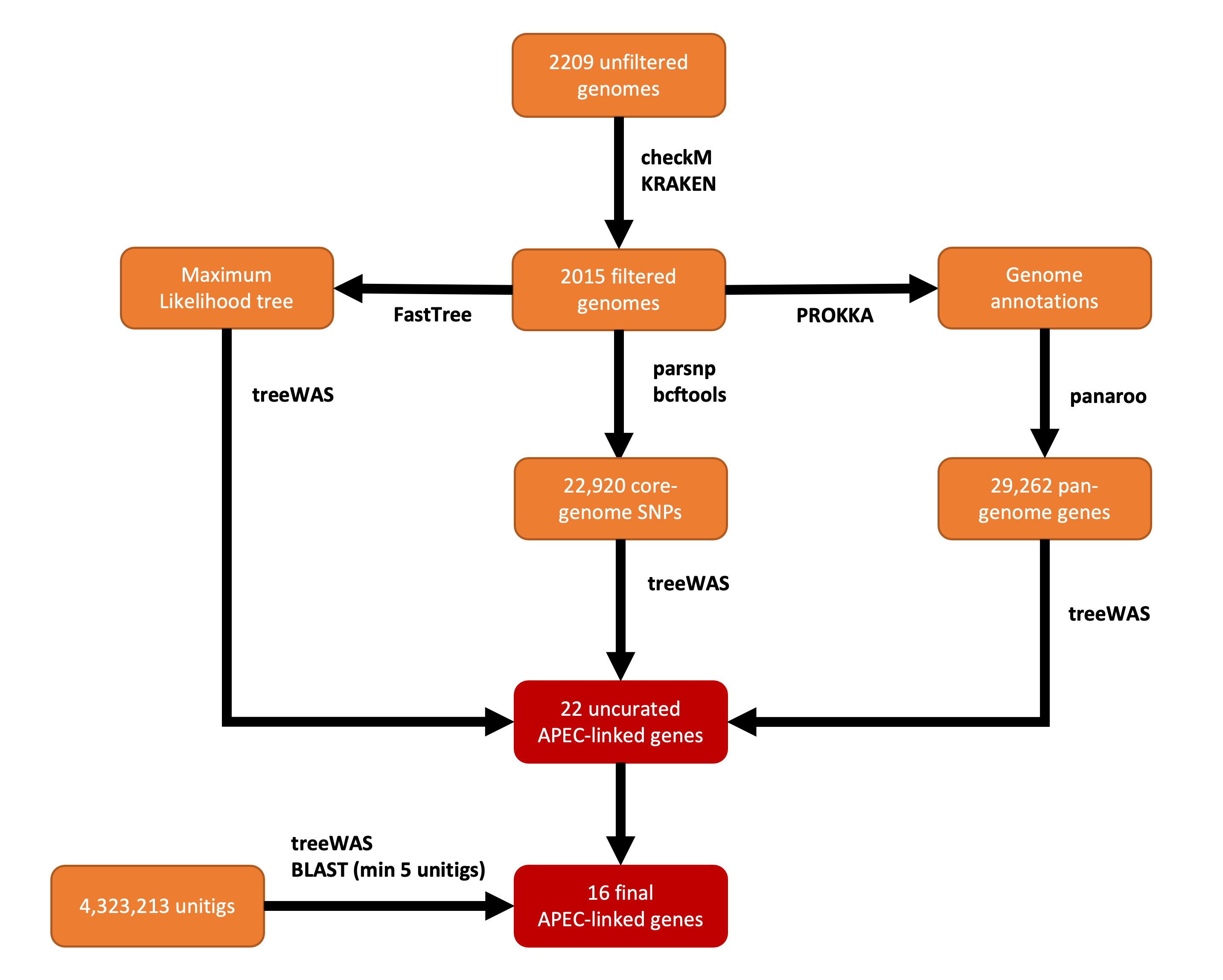

Supplement: Supplementary Figure 1 — Pipeline for the identification of genes linked to the APEC pathotype. Genome assemblies were downloaded from NCBI (unfiltered genomes), and reads data were assembled using the PATRIC web application if the genome sequences were not available. The quality of the assemblies was assessed using checkM, and low-quality genomes were filtered out based on several criteria (filtered genomes). The genomes were annotated using PROKKA (Genes annotation) and a Maximum Likelihood tree was constructed with FastTree. For the gene-based GWAS, a gene presence/absence matrix was produced with Panaroo, and treeWAS was used to identify significant genes associated with APEC isolates. Candidate genes with a prevalence > 50% in APEC and APEC vs. AFEC prevalence ratio > 1 were selected. For the SNP-based GWAS, parsnp was used to align the strains and generate a VCF file of SNPs, which were filtered and processed using bcftools. Significant SNPs associated with APEC isolates were identified using treeWAS. A unitig-based GWAS was conducted to further validate the significant genes identified from gene-based and SNP-based GWAS. The unitigs were generated and assessed for association with APEC phenotype, and the significant unitigs were aligned to the candidate genes using BLASTN. Final candidates were selected by retaining only the genes with at least 5 aligned unitigs. [file Image_1.JPEG]

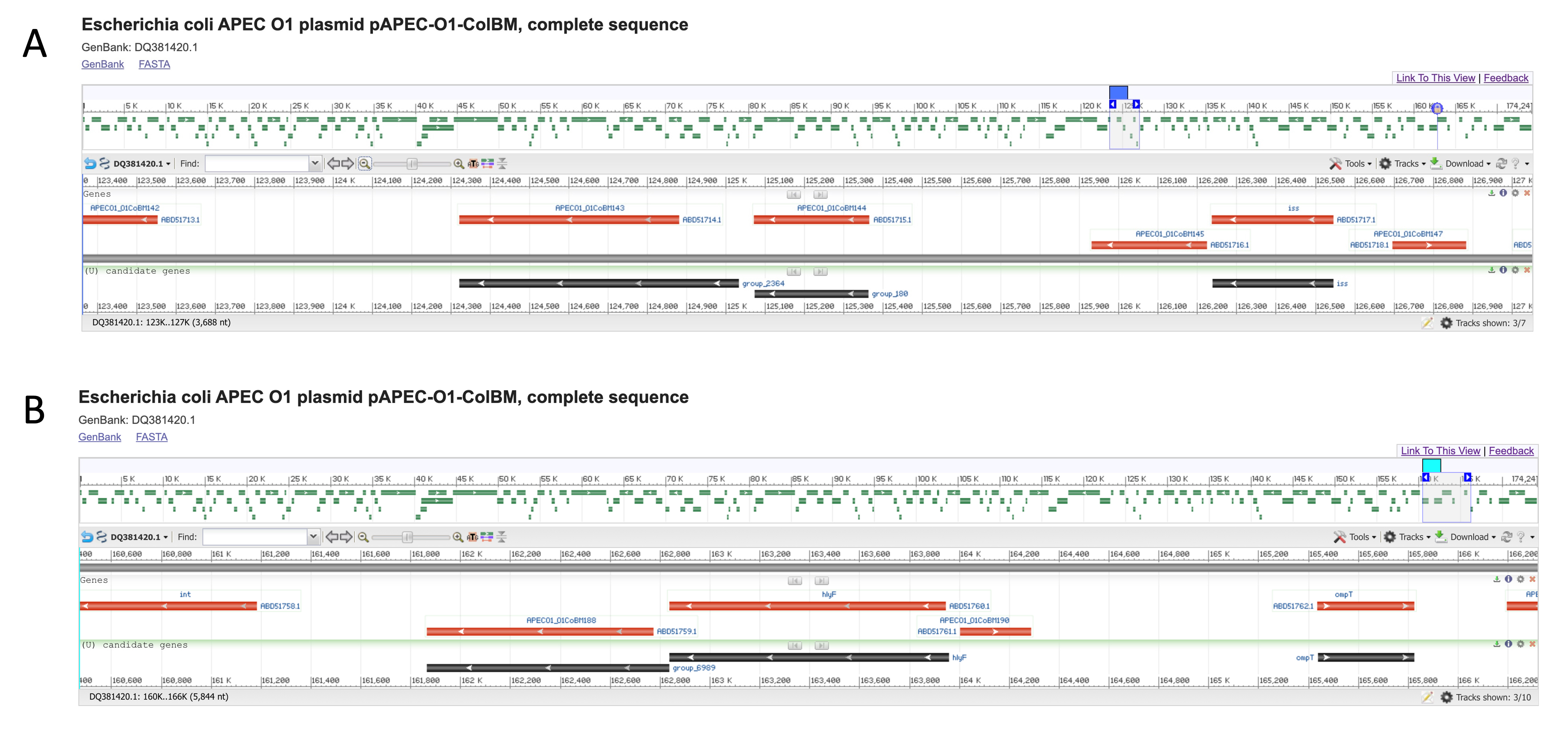

Supplement: Supplementary Figure 2 — Genomic locations of candidate genes with generic gene names. Each panel contains two tracks from top to bottom: genes and candidate genes. (A) Locations of group_2364 and group_180 on the ColV plasmid from the APEC O1 strain with respect to other candidate genes; (B) Locations of group_6989 on the ColV plasmid from the APEC O1 strain with respect to other candidate genes. [file Image_2.JPEG]
